# Pathogenic Potential and Antimicrobial Resistance Trends in Acinetobacter Species

**DOI:** 10.7759/cureus.114090

**Published:** 2026-08-06

**Authors:** Shweta D Lade, Shivaji T Mohite, Satish Patil

**Affiliations:** 1 Department of Microbiology, Krishna Vishwa Vidyapeeth (Deemed to be University), Karad, IND

**Keywords:** acinetobacter baumannii, antimicrobial resistance, antimicrobial stewardship, carbapenem resistance, nosocomial infections, pathogenicity

## Abstract

*Acinetobacter baumannii *has emerged as one of the most potent multidrug-resistant Gram-negative bacteria causing healthcare-associated illnesses worldwide. Its exceptional ability to thrive in hospital settings, acquire antibiotic resistance determinants, build biofilms, and elude host immune responses has contributed to its growing therapeutic relevance. The fast rise of multidrug-resistant (MDR), extensively drug-resistant (XDR), and carbapenem-resistant *A. baumannii* has significantly reduced available therapeutic choices while increasing morbidity and mortality. This narrative review summarizes our present understanding of* A. baumannii* epidemiology, pathogenicity, virulence factors, antimicrobial resistance mechanisms, clinical symptoms, diagnostic techniques, antimicrobial stewardship, and emerging therapy efforts. Continuous surveillance, rapid molecular diagnosis, the development of innovative antimicrobial medicines, and efficient infection prevention methods are critical for lowering the global burden of *A. baumannii* infections and improving patient outcomes.

## Introduction and background

*A. baumannii *is acknowledged worldwide as a primary source of infections linked to healthcare and has become one of the most clinically important opportunistic pathogens. It is a participant in the genus *Acinetobacter*, which contains aerobic, non-motile, Gram-negative coccobacilli that can survive under harsh environmental conditions. The organism's exceptional capacity to last on hospital surfaces, develop antibiotic resistance, and spread efficiently within healthcare facilities has made it an important public health problem [1,2]. Because patients in critical condition and with impaired immune systems are more vulnerable in the Intensive Care Unit, hospital-acquired infections caused by *A. baumannii* are more prevalent there. The pathogen is linked to a wide range of infections, such as meningitis, bloodstream infections, urinary tract infections, surgical site infections, wound infections, and ventilator-associated pneumonia. Its capacity to endure on environmental surfaces and medical equipment greatly increases the risk of transmission and outbreaks in hospital settings [1,3]. Multidrug-resistant *A. baumannii* is becoming more common, which presents significant difficulties for contemporary clinical management. Numerous mechanisms, including the production of β-lactamases and carbapenemases, activation of efflux pumps, alteration of target sites, decreased membrane permeability, and horizontal gene transfer, contribute to the growth of resistance. The organism can withstand antimicrobial treatment thanks to these resistance mechanisms and contributes to increased morbidity, mortality, and healthcare costs [4,5].

## Review

Methods

A thorough literature search was carried out to find relevant papers on the pathogenicity, virulence mechanisms, antibiotic resistance, and clinical importance of *Acinetobacter* species. Electronic databases such as PubMed and Google Scholar were searched for peer-reviewed publications written in English. The search approach included combinations of the following keywords: "*Acinetobacter baumannii*", *"Acinetobacter *species", "antimicrobial resistance", "multidrug resistance", "pathogenesis", "virulence factors", "hospital-acquired infections", "nosocomial infections", and "clinical management".

Original research articles, narrative reviews, systematic reviews, meta-analyses, clinical studies, surveillance reports, and relevant guidelines describing the epidemiology, pathogenic mechanisms, antimicrobial resistance trends, diagnosis, treatment, and infection prevention strategies of *Acinetobacter* species were eligible for inclusion. Studies unrelated to the review aims, duplicate publications, conference abstracts without full-text access, editorials, and articles published in languages other than English were eliminated.

The selected literature was reviewed narratively, and the results were organised into thematic sections such as epidemiology, virulence factors, antimicrobial resistance mechanisms, clinical manifestations, therapeutic approaches, and emerging strategies for* Acinetobacter* infection prevention and management. The collected data were synthesized to provide a comprehensive overview of the current understanding of *Acinetobacter* species and their growing clinical importance. The characteristics and key findings of the included studies are summarized in Table 1.

**Table 1:** Summary of key publications discussed in this narrative review Overview of the studies included in this review and their principal findings regarding the microbiology, virulence, epidemiology, antimicrobial resistance, clinical manifestations, and therapeutic management of *Acinetobacter baumannii*

Sr. No.	Author (Year)	Study Type	Key Findings
1	Saad et al., 2018 [1]	Review Article	Described Acinetobacter spp. as important nosocomial pathogens with increasing multidrug resistance and significant epidemiological impact.
2	Wenzler et al., 2017 [2]	Review Article	Highlighted the role of antimicrobial stewardship in controlling Acinetobacter infections and reducing resistance development.
3	Antunes et al., 2014 [3]	Review Article	Discussed the global emergence and evolution of A. baumannii as a multidrug-resistant pathogen.
4	Moubareck & Halat, 2020 [4]	Review Article	Summarized microbiological characteristics, virulence factors, and antimicrobial resistance mechanisms of A. baumannii.
5	Yehya et al., (2025) [5]	Comprehensive Review	Provided an updated overview of multidrug resistance, pathogenicity, clinical significance, and therapeutic challenges.
6	Nguyen & Joshi, 2021 [6]	Review Article	Examined carbapenem resistance mechanisms and their role in hospital-acquired infections.
7	CDC, 2022 [7]	Surveillance Report	Reported increased antimicrobial resistance and healthcare-associated infections during and after the COVID-19 pandemic.
8	Darby et al., 2024 [8]	Original Research	Demonstrated species-specific differences in virulence and antibiotic resistance development among Acinetobacter species.
9	Gordon & Wareham, 2010 [9]	Review Article	Reviewed molecular mechanisms of virulence and multidrug resistance in Acinetobacter.
10	Anstey et al., 2002 [10]	Clinical Study	Showed that community-acquired bacteremic pneumonia can be caused by diverse strains of A. baumannii in tropical regions.
11	Dubey et al., 2025 [11]	Review Article	Discussed mortality, emerging therapeutic options, and future drug targets for resistant A. baumannii infections.
12	Peleg et al., 2008 [12]	Review Article	Identified A. baumannii as a highly successful pathogen due to environmental persistence and resistance acquisition.
13	Howard et al., 2012 [13]	Review Article	Described major virulence determinants including biofilm formation, capsule production, and environmental survival.
14	Fournier & Richet, 2006 [14]	Review Article	Reviewed epidemiology, transmission dynamics, outbreak investigations, and infection-control strategies.
15	Wong et al., 2017 [15]	Review Article	Provided a comprehensive overview of clinical manifestations and pathophysiology of Acinetobacter infections.
16	Harding et al., 2018 [16]	Review Article	Explained molecular mechanisms responsible for virulence, host colonization, and persistence.
17	Lee et al., 2017 [17]	Review Article	Summarized pathogenesis, antibiotic resistance mechanisms, and potential therapeutic approaches.
18	Towner, 2009 [18]	Review Article	Highlighted the increasing global burden and epidemiology of Acinetobacter infections.
19	Richards et al., 2024 [19]	Review Article	Discussed challenges associated with difficult-to-treat and multidrug-resistant Acinetobacter infections.
20	Tacconelli et al., 2018 [20]	WHO Priority Pathogen Report	Classified carbapenem-resistant A. baumannii as a critical-priority pathogen requiring urgent development of new antimicrobial agents.

Microbiology of *Acinetobacter baumannii*

*A. baumannii* is a coccobacillus that is oxidase-negative, catalase-positive, aerobic, non-fermentative, and Gram-negative. It belongs to the Moraxellaceae family. It may survive in soil, water, and medical settings due to its remarkable environmental resilience and widespread distribution in nature [1,2]. Because of its substantial correlation with hospital-acquired infections and antibiotic resistance, *A. baumannii *is regarded as the most clinically significant species in the *Acinetobacter *genus. The organism facilitates transmission and persistence in healthcare facilities because it can survive for prolonged periods on dry surfaces, medical equipment, and hospital environments [1,3]. In terms of microbiology, *A. baumannii* thrives on common lab media, such as MacConkey agar and blood agar. Usually, colonies have a smooth, opaque, and pigment-free appearance. *Acinetobacter* species use oxidative metabolic pathways to produce energy instead of fermenting carbohydrates, in contrast to many Gram-negative bacteria [3]. The exceptional genetic flexibility of *A. baumannii *is one of its distinguishing features. The organism quickly adjusts to environmental stress and antimicrobial exposure by horizontal gene transfer, acquisition of mobile genetic elements, plasmids, insertion sequences, and resistance islands [2,4]. This genetic adaptability helps strains become resistant to several drugs and promotes survival under antimicrobial pressure. One of the main microbiological issues with *A. baumannii* is carbapenem resistance. The production of carbapenem-hydrolyzing enzymes (carbapenemases), activation of multidrug efflux pumps, alterations in membrane permeability, and mutations affecting antibiotic targets are the major mechanisms of antimicrobial resistance. These modifications complicate treatment outcomes and drastically lower therapeutic efficacy [4,5]. The intricacy of bacterial adaptability and clinical management is highlighted by recent research that indicates differences across *Acinetobacter* species impact patterns of virulence and resistance development [6].

Expanded Resistance Mechanisms

In addition to carbapenemase production, *A. baumannii* employs several complementary mechanisms to resist antimicrobial therapy. Overexpression of resistance-nodulation-division (RND) efflux pumps, particularly AdeABC, AdeFGH, and AdeIJK, actively exports multiple classes of antibiotics, including aminoglycosides, fluoroquinolones, tetracyclines, and tigecycline. Alterations or loss of outer membrane proteins such as CarO decrease membrane permeability, thereby limiting antibiotic uptake. Furthermore, mutations in the gyrA and parC genes confer fluoroquinolone resistance, whereas aminoglycoside-modifying enzymes reduce susceptibility to aminoglycosides. Horizontal gene transfer through plasmids, transposons, integrons, and resistance islands facilitates dissemination of resistance determinants among clinical isolates, while biofilm formation further enhances antimicrobial tolerance and promotes persistent infections [5,6].

Pathogenicity of *Acinetobacter baumannii*

The pathogenicity of *A. baumannii* is influenced by several virulence factors that promote colonization, invasion, persistence, immune evasion, and resistance to antimicrobial therapy. These characteristics make *A. baumannii* a highly successful opportunistic pathogen. Its pathogenicity is driven by multiple virulence factors that promote colonization, tissue invasion, persistence, immune evasion, and survival in healthcare environments, thereby contributing to healthcare-associated infections [3,7]. Among the most crucial pathogenicity mechanisms is the formation of biofilms. Biofilms are collections of bacteria covered in a protective extracellular matrix that sticks to both biotic and abiotic surfaces. Biofilm development enables *A. baumannii *to flourish on ventilators, catheters, and medical equipment in addition to protecting bacterial cells from medications and host immunological responses [3,5]. Another essential element is the development of capsules, which prevent bacterial cells from dissolution and immune-mediated death. The capsule enhances persistence within the host and increases resistance to phagocytosis [7]. Additionally, *A. baumannii *is highly resilient to environmental stresses, which allows for prolonged life in medical environments. This environmental resilience increases the likelihood of transmission and leads to recurrent outbreaks [1,2]. The bacterium often causes infections of the bloodstream, urinary tract, or wounds, ventilator-associated pneumonia, and sepsis, especially in very sick and immunocompromised individuals [3,8]. Reports of community-acquired infections in tropical locations demonstrate that *A. baumannii*'s pathogenic potential is not restricted to hospital settings [9]. The COVID-19 pandemic further highlighted the clinical importance of antimicrobial-resistant bacteria, particularly *A. baumannii*. Increased empirical antibiotic use, prolonged intensive care unit admissions, mechanical ventilation, and healthcare system challenges during the pandemic contributed to higher rates of antimicrobial resistance and healthcare-associated infections [7,10].

Biofilm Formation

Biofilm production is a crucial virulence factor for *A. baumannii*. Biofilms improve adhesion to abiotic surfaces such as ventilators, catheters, and other medical devices. Bacteria lodged in biofilms are resistant to antibiotics, disinfectants, and host immunological defences, resulting in recurrent infections and treatment failure [4].

Capsule Production

The polysaccharide capsule defends bacterial cells against phagocytosis and complement-mediated death. Capsule development improves bacterial survival within the host, aids in immune evasion, and boosts overall virulence by encouraging persistence after infection [12,13].

Iron Acquisition Systems

Iron acquisition processes allow *A. baumannii* to receive vital nutrients in iron-deficient host settings. These specialised mechanisms promote bacterial proliferation, colonisation, and persistence after infection, greatly contributing to pathogenicity [3,4].

Environmental Persistence

*A. baumannii's* exceptional capacity to survive in severe environments, such as prolonged desiccation and exposure to hospital disinfectants, contributes greatly to its spread within healthcare institutions and facilitates nosocomial outbreaks. This environmental resilience is a major contributing element to its emergence as a major multidrug-resistant healthcare-associated infection [16,18].

Host immune response to* Acinetobacter baumannii*

The interaction between *A. baumannii* and the human immune system is critical for disease severity and clinical consequences. Toll-like receptors recognise pathogen-associated molecular patterns during infection, activating the innate immune response and producing pro-inflammatory cytokines like tumor necrosis factor α (TNF-α), IL-1β, and IL-6. The principal defence mechanisms against *A. baumannii* are neutrophils and macrophages; nevertheless, the organism uses a variety of immune-evasion techniques, such as capsule synthesis, biofilm formation, and resistance to complement-mediated death. Excessive inflammatory reactions may cause tissue damage and increase death in severely unwell individuals. A greater understanding of host-pathogen interactions should aid in the development of immunomodulatory treatments and vaccines [15,16].

*Acinetobacter baumannii-*related infections

One of the most crucial opportunistic bacteria in the world today that causes illnesses related to healthcare is *A. baumannii.* Its clinical importance has grown as a result of its ability to colonize humans, survive in hospital environments, and develop resistance to many antibiotic treatments [1,2]. Immunocompromised individuals, individuals in severe condition, Intensive Care Unit admissions, and those receiving long-term antimicrobial treatment are more likely to get *A. baumannii* infections [8]. Ventilator-associated pneumonia is among the most prevalent signs of an* A. baumannii *infection. Bacterial colonization and biofilm development are facilitated by mechanical ventilation, a place that enables the organism to infiltrate the lower respiratory tract.* A. baumannii-*induced hospital-acquired pneumonia is linked to extended hospital stays, increased medical expenses, and higher death rates [3,8]. Bacteremia, or bloodstream infection, is another clinically significant illness. Patients who use central venous catheters and other invasive medical equipment often develop *A. baumannii *bacteremia, which is linked to severe systemic inflammatory reactions and septic consequences. Multidrug-resistant forms of bloodstream infections frequently lead to poor patient outcomes and few therapeutic options [1,7].

*Urinary Tract Infections* 

Urinary tract infections caused by *A. baumannii* are often associated with extended hospital stays and also the installation of catheters. Urinary catheter colonization promotes bacterial growth and biofilm development, which increases antibiotic resistance and causes recurrent infections [3]. Additionally, the organism has a major impact on the surgical site and wound infections, particularly in individuals who have been traumatized, have burns, or have difficulties following surgery. These infections raise the possibility of systemic dissemination, additional problems, and delayed recovery [3]. Additionally, *A. baumannii* has been connected to meningitis, especially after head trauma and neurosurgical treatments. Due to decreased antibiotic penetration and growing resistance mechanisms, central nervous system infections following surgery caused by resistant bacteria are still challenging to treat [7]. *A. baumannii* is largely known as a pathogen acquired in hospitals, although it can also cause other infections.

Community-Acquired Infections

There are recorded instances of community-acquired bacteremic pneumonia, especially in tropical areas, suggesting that host-related and environmental variables may play a role in infection outside of clinical settings [9].* A. baumannii *has been reported to cause infections in the local area, especially in tropical areas, in addition to other hospital-acquired diseases. This implies that the transfer of this bacterium may be influenced by elements outside of healthcare environments.

Bloodstream Infection

*A. baumannii *bloodstream infections are among the most serious illnesses linked to healthcare and are linked to significant morbidity and mortality globally. The pathogen's capacity to persist in hospital settings, colonise susceptible people, and acquire resistance to several antimicrobial treatments has made it more significant [1,2]. Hospitalized patients, particularly those admitted to Intensive Care Units on long-term antibiotic therapy; patients undergoing invasive medical operations; and immunocompromised populations are the main groups at risk for *A. baumannii *bloodstream infections. Extended hospital stays, ventilation with a machine, central venous catheter use, surgical procedures, and underlying chronic diseases are frequently linked to bacteremia [1,8]. Bacterial entry into the bloodstream follows colonization of the skin, respiratory system, medical equipment, or environmental surfaces in the pathophysiology of bloodstream infection. The organism has many virulence factors that support bacteria surviving and spread throughout the host, including biofilm formation, capsule development, outer membrane proteins, and iron acquisition systems [1,2]. In bloodstream infections linked to catheter use, biofilm growth is especially crucial. Biofilms enable sustained colonization of intravascular devices and raise the danger of recurrent infection by protecting bacterial cells from antibacterial drugs as well as host immunity defenses [3,5].

Antibiogram of* Acinetobacter* species

A cumulative laboratory report that describes the antibiotic susceptibility patterns of bacterial isolates in a medical environment over a certain time period is called an antibiogram. Antibiogram analysis is essential for directing empirical treatment, tracking antimicrobial resistance trends, and bolstering antimicrobial stewardship initiatives in *Acinetobacter* species [11]. *A. baumannii *is widely recognised for its ability to rapidly develop resistance to several antibiotic classes, making treatment more challenging. β-lactamase synthesis, carbapenemase activity, efflux pumps, target alteration, reduced membrane permeability, and horizontal gene transfer are some of the pathways that lead to resistance [12,13]. Hospital infection outcomes are greatly impacted by the introduction of strains of *A. baumannii* that are carbapenem-resistant, extensively drug-resistant, and multidrug-resistant [14,15].

Β-Lactam Antibiotics

Bacterial cell wall production is disrupted by β-lactam antibiotics. Nevertheless, *Acinetobacter* frequently generates β-lactamases that diminish the efficacy of numerous cephalosporins and penicillins [13].

Carbapenems

In the past, carbapenems like imipenem and meropenem were thought to be crucial treatments for serious *Acinetobacter* infections. However, the prevalence of carbapenem resistance caused by OXA-type carbapenemases and other mechanisms has increased [12,13].

Aminoglycosides

By attaching to bacterial ribosomes, substances like amikacin and gentamicin prevent the synthesis of proteins. Some isolates may still be active despite the increasing prevalence of resistance [11].

Fluoroquinolones

Fluoroquinolones inhibit DNA replication-related enzymes. Resistance is often caused by target mutations and efflux mechanisms, which reduces treatment utility [13].

Tetracycline Derivatives

Minocycline and tigecycline are commonly assessed in treatment plans and may show action against certain isolates that can withstand multiple drugs [11].

Polymyxins

The bacterial outer membrane is disrupted by colistin (polymyxin E) and polymyxin B, which are frequently saved for challenging multidrug-resistant diseases. Because of toxicity concerns, its usage necessitates close clinical monitoring [11,13].

Species

At the genus level, *Acinetobacter* can be assumed to be Gram-negative, catalase-positive, oxidase-negative, non-motile, non-fermenting coccobacilli. Nevertheless, the organisms are frequently misidentified as Gram-positive because they are difficult to de-stain. *Acinetobacter* cannot be distinguished from other non-fermenting Gram-negative bacteria using a conclusive metabolic test. The ability of the mutant *A. baylyi *strain BD413 trpE27 to be changed to a wild-type phenotype by crude DNA of any *Acinetobacter* species is a method that is frequently used to identify to the genus level. The 28 phenotypic tests that are now available have been shown to be 95.6% successful in detecting *Acinetobacter* generated from human skin at the species level. However, more recent genomic strains of *Acinetobacter* have proven difficult to identify using phenotypic tests alone.

Because phenotypic methods have limited ability to distinguish closely related *Acinetobacter* species, molecular diagnostic techniques have become important for accurate species identification and epidemiological investigations.

Accurate species-level identification of *Acinetobacter* has been greatly improved by molecular diagnostic methods. Techniques such as amplified ribosomal DNA restriction analysis (ARDRA), amplified fragment length polymorphism (AFLP), ribotyping, analysis of the 16S-23S rRNA intergenic spacer region, and sequencing of the rpoB gene provide higher discriminatory power than conventional phenotypic methods. These approaches improve identification accuracy, facilitate epidemiological investigations, and assist in detecting genetically related strains during hospital outbreaks [13].

Antimicrobial stewardship and resistance control

The significance of programs for antimicrobial stewardship has been highlighted by the rise of antibiotic resistance among *Acinetobacter* species. In order to lessen the development of resistance and enhance patient outcomes, stewardship focuses on proper antibiotic selection, optimum dose, susceptibility-guided therapy, infection prevention strategies, and surveillance [11,16]. Strict hand hygiene, environmental disinfection, surveillance cultures, and antibiotic monitoring programs can reduce healthcare-associated transmission [17,18].

Diagnostic approaches

Early and accurate detection of* A. baumannii* infections is critical for the initiation of effective antibiotic therapy and infection-control measures. Bacterial culture, biochemical identification, and antibiotic susceptibility testing are the three main components of conventional laboratory diagnosis. In addition to conventional culture and biochemical identification, modern diagnostic methods such as matrix-assisted laser desorption/ionization-time of flight (MALDI-TOF) mass spectrometry, polymerase chain reaction (PCR)-based assays, and whole-genome sequencing enable rapid species identification and detection of antimicrobial resistance genes. These techniques improve diagnostic accuracy, shorten turnaround time, and support outbreak investigations and infection-control measures in healthcare settings [17].

Vaccine development and prevention strategies

Despite substantial study, no approved vaccination for *A. baumannii* is currently available. Several vaccine candidates aimed at outer membrane proteins, capsular polysaccharides, outer membrane vesicles, and biofilm-associated proteins have demonstrated immunogenicity and protective effectiveness in preclinical trials. Advances in reverse vaccinology and genomic technology have hastened the discovery of new vaccine targets. The development of an effective vaccine, together with antimicrobial stewardship, surveillance systems, and stringent infection-prevention techniques, could provide a long-term strategy for lowering the global incidence of multidrug-resistant *A. baumannii* infections [17,20].

Clinical significance

*Acinetobacter* has become a major worldwide health issue because of the rise in resistant variants. To improve infection control and avoid the establishment of resistant bacteria, sensible antibiotic administration and ongoing antibiogram monitoring are still crucial [19,20].

Epidemiology

Numerous epidemiological studies have demonstrated the global spread of *A. baumannii* in healthcare settings, where it has become a major cause of hospital-associated outbreaks because of its environmental persistence and multidrug-resistant nature. Individual *A. baumannii *strains have been known to spread in a variety of ways, including outbreaks in several hospitals within a city, outbreaks in several cities within a nation, and outbreaks in hospitals across various nations. Thirty-four distinct *A. baumannii* genotypes were found in 46 UK hospitals during a survey conducted in 1999-2001. These genotypes were grouped into 10 distinct clusters, with certain strains typically associated with specific hospitals. Two carbapenem-resistant *A. baumannii* lineages (SE clone and OXA-23 clone) that are only responsive to colistin and tigecycline became common in more than 40 UK hospitals between 2003 and 2006. A survey involving 130 hospitals reported the widespread detection of carbapenem-resistant *A. baumannii*, demonstrating its transition from an infrequently isolated organism to an endemic and, in some healthcare settings, epidemic pathogen [18]. Random amplified polymorphic DNA, pulsed-field gel electrophoresis, and polymerase chain reaction-based sequence typing were used to identify a variety of clusters in European hospitals; nonetheless, three main European lineages were found. Several isolates from a single hospital typically belonged to the same clone, just like in the UK [18].

Discussion

The rising incidence of multidrug- and carbapenem-resistant *Acinetobacter* species poses a significant challenge to modern healthcare systems. The organism's unusual combination of virulence characteristics, environmental persistence, and genetic adaptation allows for successful colonisation and transmission in healthcare contexts. Continued surveillance, stewardship measures, and the development of new therapeutic techniques are critical.

Limitations

This review has a few restrictions. First, the reviewed literature differed significantly in terms of design, technique, sample size, geographic distribution, and reporting quality, which may have introduced variability in the interpretation of findings. Second, because this is a narrative review, no quantitative meta-analysis was conducted, restricting the capacity to compare outcomes statistically. Publication bias may have influenced the available evidence, as research with substantial or unique findings is more likely to be published than those with negative or inconclusive results. Furthermore, the quickly changing epidemiology and antibiotic resistance patterns of *A. baumannii* may restrict the long-term use of some findings. The advent of new resistance mechanisms, as well as regional variations in antimicrobial sensitivity, highlight the importance of ongoing surveillance and regular literature updates. Furthermore, variations in infection-control procedures and antimicrobial stewardship policies among healthcare institutions may limit the generalisability of the reviewed findings [15,19].

Recommendations

Antimicrobial stewardship programs at healthcare facilities should be strengthened in order to encourage rational antibiotic usage and limit the spread of multidrug-resistant *A. baumannii*. Rigorous infection-prevention and control methods, such as stringent hand hygiene, environmental cleaning, isolation of colonised or infected patients, and proper sterilisation of medical equipment, should be routinely implemented. To improve clinical outcomes, routine antibiotic resistance surveillance and susceptibility-guided therapy should be encouraged. Healthcare professionals should receive ongoing education on antimicrobial stewardship principles and infection control measures. Furthermore, interdisciplinary coordination among physicians, microbiologists, chemists, and infection-control teams is critical for early diagnosis, effective management, and prevention of *A. baumannii*-caused hospital outbreaks [14,15].

Future Research Directions

Future research should concentrate on the discovery and clinical testing of new antimicrobial compounds active against multidrug-resistant and extensively drug-resistant *A. baumannii*. New therapeutic techniques, such as cefiderocol-based regimens, sulbactam-durlobactam combos, anti-biofilm medicines, bacteriophage therapy, antimicrobial peptides, and immunotherapeutic strategies, require more exploration. Research should also prioritise genomic surveillance to track the evolution and spread of resistance genes, as well as the use of whole-genome sequencing to better understand transmission dynamics and epidemic epidemiology. The development of quick molecular diagnostic methods capable of reliably detecting resistance determinants and virulence factors would allow for more timely diagnosis and focused treatment. Furthermore, multicenter prospective clinical trials are required to assess the long-term efficacy of new treatment regimens, optimise combination medications, and improve outcomes in patients with severe *A. baumannii* infections [19,20].

## Conclusions

*A. baumannii *remains one of the most difficult healthcare-associated infections due to its amazing environmental survival, diverse virulence factors, and emerging antibiotic resistance pathways. Despite tremendous breakthroughs in understanding its biology, numerous major issues remain unanswered. Rapid and accurate detection of resistant bacteria remains limited in many healthcare settings, delaying necessary antibiotic medication and infection-control strategies. The ongoing development and global spread of carbapenemases, efflux pump-mediated resistance, biofilm-associated tolerance, and horizontally acquired resistance genes continue to diminish the efficacy of existing antimicrobial drugs and complicate clinical management. Although newer therapeutic options, such as cefiderocol and sulbactam-based therapies, are promising for multidrug-resistant infections, emerging resistance to these agents highlights the critical need for ongoing antimicrobial stewardship, surveillance, and the development of novel therapeutic approaches. Future research should prioritize rapid molecular diagnostic methods, continuous genomic surveillance, better understanding of resistance evolution, combination therapy optimisation, and the development of novel treatment strategies to improve clinical outcomes and reduce the global burden of *A. baumannii *infections. Future efforts should concentrate on developing quick molecular diagnostic technologies, identifying novel antibiotic targets, optimising combination medicines, and implementing effective antimicrobial stewardship and infection prevention programs. Continued study into resistance evolution and creative therapeutic approaches will be necessary to enhance clinical outcomes and minimise the global burden of *A. baumannii* infections.
